# Timing of interval debulking surgery and postoperative chemotherapy after neoadjuvant chemotherapy in advanced epithelial ovarian cancer: a multicenter real-world study

**DOI:** 10.1186/s13048-023-01164-8

**Published:** 2023-06-27

**Authors:** Xingyu Liu, Yingjun Zhao, Xiaofei Jiao, Yang Yu, Ruyuan Li, Shaoqing Zeng, Jianhua Chi, Guanchen Ma, Yabing Huo, Ming Li, Zikun Peng, Jiahao Liu, Qi Zhou, Dongling Zou, Li Wang, Qingshui Li, Jing Wang, Shuzhong Yao, Youguo Chen, Ding Ma, Ting Hu, Qinglei Gao

**Affiliations:** 1 Department of Gynecological Oncology, Tongji Hospital, Tongji Medical College, Huazhong University of Science and Technology, Wuhan, China; 2 National Clinical Research Center for Obstetrics and Gynecology, Cancer Biology Research Center (Key Laboratory of the Ministry of Education), Tongji Hospital, Tongji Medical College, Huazhong University of Science and Technology, Wuhan, China; 3 Cancer Hospital, National Cancer Center, Chinese Academy of Medical Sciences and Peking Union Medical College, Beijing, China; 4 Department of Gynecologic Oncology, Chongqing University Cancer Hospital, Chongqing, China; 5 Chongqing Key Laboratory of Translational Research for Cancer Metastasis and Individualized Treatment, Chongqing University Cancer Hospital, Chongqing, China; 6 Key Laboratory for Biorheological Science and Technology of Ministry of Education (Chongqing University), Chongqing University Cancer Hospital, Chongqing, China; 7 Department of Cancer Biology Immunotherapy, The Affiliated Cancer Hospital of Zhengzhou University and Henan Cancer Hospital, Zhengzhou, Henan China; 8 Department of Gynecologic Oncology, Shandong Cancer Hospital and Institute, Jinan, Shandong China; 9 Hunan Clinical Research Center in Gynecologic Cancer, Hunan Cancer Hospital and The Affiliated Cancer Hospital of Xiangya School of Medicine, Central South University, Changsha, Hunan China; 10 Department of Gynecologic Cancer, Hunan Cancer Hospital and The Affiliated Cancer Hospital of Xiangya School of Medicine, Central South University, Changsha, Hunan China; 11 Department of Obstetrics and Gynecology, the First Affiliated Hospital, Sun Yat-sen University, Guangzhou, Guangdong China; 12 Department of Gynecology & Obstetrics, the First Affiliated Hospital of Soochow University, Suzhou, Jiangsu Province China; 13 Cancer Biology Research Center (Key Laboratory of the Ministry of Education), Tongji Hospital, Tongji Medical College, Huazhong University of Science and Technology, 1095 Jiefang Ave, Wuhan, 430000 China

**Keywords:** Neoadjuvant chemotherapy, Time to interval debulking surgery, Time to postoperative adjuvant chemotherapy, Advanced epithelial ovarian cancer, Prognosis

## Abstract

**Background:**

To investigate the prognostic relevance of the time to interval debulking surgery (TTS) and the time to postoperative adjuvant chemotherapy (TTC) after the completion of neoadjuvant chemotherapy (NACT).

**Methods:**

A retrospective real-word study included 658 patients with histologically confirmed advanced epithelial ovarian cancer who received NACT at seven tertiary hospitals in China from June 2008 to June 2020. TTS was defined as the time interval from the completion of NACT to the time of interval debulking surgery (IDS). TTC was defined as the time interval from the completion of NACT to the initiation of postoperative adjuvant chemotherapy (PACT).

**Results:**

The median TTS and TTC were 25 (IQR, 20–29) and 40 (IQR, 33–49) days, respectively. Patients with TTS > 25 days were older (55 vs. 53 years, *P* = 0.012) and received more NACT cycles (median, 3 vs. 2, *P* = 0.002). Similar results were observed in patients with TTC > 40 days. In the multivariate analyses, TTS and TTC were not associated with PFS when stratified by median, quartile, or integrated as continuous variables (all *P* > 0.05). However, TTS and TTC were significantly associated with worse OS when stratified by median (*P* = 0.018 and 0.018, respectively), quartile (*P* = 0.169, 0.014, 0.027 and 0.012, 0.001, 0.033, respectively), or integrated as continuous variables (*P* = 0.018 and 0.011, respectively). Similarly, increasing TTS and TTC intervals were associated with a higher risk of death (*P*_*trend*_ = 0.016 and 0.031, respectively) but not with recurrence (*P*_*trend*_ = 0.103 and 0.381, respectively).

**Conclusion:**

The delays of IDS and PACT after the completion of NACT have adverse impacts on OS but no impacts on PFS, which indicates that reducing delays of IDS and PACT might ameliorate the outcomes of ovarian cancer patients treated with NACT.

**Supplementary Information:**

The online version contains supplementary material available at 10.1186/s13048-023-01164-8.

## Background

Ovarian cancer is a notorious gynecological malignancy with an abysmal prognosis, of which the 5-year survival rate is 35% [1, 2]. In 2020, the estimated number of new cases and deaths from ovarian cancer in China was approximately 54,709 and 39,894, respectively, ranking first in the world [3]. Neoadjuvant chemotherapy followed by interval debulking surgery (NACT-IDS) is an alternative to primary debulking surgery (PDS) recommended by National Comprehensive Cancer Network for advanced ovarian cancer patients [4]. In several randomized controlled trials (RCTs), NACT-IDS significantly reduced postoperative complications and mortality, and patients treated with NACT-IDS achieved a survival that was non-inferior to patients treated with PDS [5–8]. For advanced patients with extensive metastases or unresectable disease, NACT provided the opportunity for surgery and significantly ameliorated the optimal resection rate [5–8]. Thereby, the utilization of NACT-IDS has been growing in the past decade [9, 10]. However, various controversies related to NACT have ensued. One of the controversies is the appropriate timing of IDS and postoperative adjuvant chemotherapy (PACT) after the completion of NACT.

In clinical practice, the consensus is to perform IDS after recovery from neutropenia. Besides, various non-clinical reasons may bring about delays of IDS in real-world settings. The previous findings are conflicting as to whether this delay has a detrimental effect on survival in ovarian cancer. Some studies showed that IDS should be performed within 25–28 days of completion of NACT [11, 12], while another study revealed that the timing of IDS was not significantly associated with prognosis [13]. As for PACT, some studies found that delayed PACT worsened survival [14–16], but another study reported no correlation between a delay in PACT and survival [17]. Whether the delay of PACT would impact survival has not been determined.

In current large-scale RCTs of NACT for advanced epithelial ovarian cancer, optimal timing of IDS and PACT has not been thoroughly addressed. At present, no guidelines specify a recommended timing of IDS and PACT after NACT. Besides, prospective or randomized controlled studies on this topic are unethical and tough to execute in practice. Herein, we performed a real-world study to explore the timing of IDS and PACT in advanced ovarian cancer patients who had received NACT.

## Results

### Clinical characteristics

A total of 658 patients who had received NACT plus IDS and PACT were enrolled in this study. As shown in Table 1, the median age was 54 (range, 48–61) years. Two hundred and twenty-eight (34.7%) patients had comorbidities and 537 (81.6%) patients developed ascites. Most patients had serous and high-grade (84.3%, 84.0%, respectively) ovarian cancer. Five hundred and thirty-seven (81.6%) patients achieved optimal surgical resection, of which 297 (45.1%) patients underwent complete surgical resection. The median number of NACT cycles was 2 (IQR, 2–3) and the median number of total cycles (the total number of cycles of both NACT and PACT) was 7 (IQR, 6–9). The median TTS and TTC were 25 (range, 8–174) days, and 40 (range, 16–187) days, respectively.

**Table 1:** Clinical characteristics of patients

	Total (N = 658)
**Age (years, median (IQR))**	54.00 (48.00, 61.00)
**Menopause**	
Yes	406 (61.7)
No	231 (35.1)
Unknown	21 (3.2)
**BMI (kg/m**^**2**^, **median (IQR))**	22.52 (20.55, 24.63)
**Comorbidity**	
Yes	228 (34.7)
No	430 (65.3)
**Type**	
Serous	555 (84.3)
Other	103 (15.7)
**Grade**	
High	553 (84.0)
Other	105 (16.0)
**FIGO stage**	
IIIC	423 (64.3)
IV	235 (35.7)
**CA125 (U/ml, median (IQR))**	1395.70 (638.50, 3180.00)
**Ascites**	
Yes	537 (81.6)
No	114 (17.3)
Unknown	7 (1.1)
**Operation time (mins, median (IQR))**	203.00 (160.00, 280.00)
**Blood loss (ml, median (IQR))**	300.00 (200.00, 600.00)
**Surgical procedure**	
Upper abdominal surgery	75 (11.4)
Bowel resection	90 (13.7)
Lymphadenectomy	343 (52.1)
**Residual disease**	
R0	297 (45.1)
R1	240 (36.5)
R2	121 (18.4)
**Postoperative complication**	
Yes	333 (50.6)
No	325 (49.4)
**Hospitalization (days, median (IQR))**	16 (14, 20)
**NACT cycles (median (IQR))**	2 (2, 3)
**Chemotherapy regimen**	
Platinum + Paclitaxel	646 (98.2)
Other	12 (1.8)
**Total cycles (median (IQR))**	7 (6, 9)

Abbreviation: IQR, Interquartile range; BMI, Body mass index; FIGO, International Federation of Gynecology and Obstetrics; CA125, Cancer antigen 125; NACT, Neoadjuvant chemotherapy; Total cycles, the total number of cycles of both neoadjuvant chemotherapy and postoperative adjuvant chemotherapy

The clinical characteristics of patients with different durations of TTS (≤ 25 days vs. > 25 days) were compared in Supplementary Table S2. Three hundred and seventy-six (57.1%) patients were treated with IDS within 25 days after the completion of NACT, and the other 282 (42.9%) patients received IDS exceeding 25 days after the completion of NACT. Patients with TTS > 25 days were older (55 years vs. 53 years, *P* = 0.012) and administrated with more cycles of NACT (median 3 vs. 2, *P* = 0.002) than patients with TTS ≤ 25 days. Other clinical characteristics were of no significant difference between these two groups.

Three hundred and forty-six (52.6%) patients and 312 (47.4%) patients were treated with PACT within and exceeding 40 days after the completion of NACT, respectively. Besides, 336 (51.1%) patients and 322 (48.9%) patients received PACT within and exceeding 13 days after IDS, respectively. We divided the patients into two groups according to the median TTC interval (TTC ≤ 40 days vs. TTC > 40 days). Comparisons between these two groups were performed (Supplementary Table S3).

### Survival analysis based on TTS

**Fig. 1:**
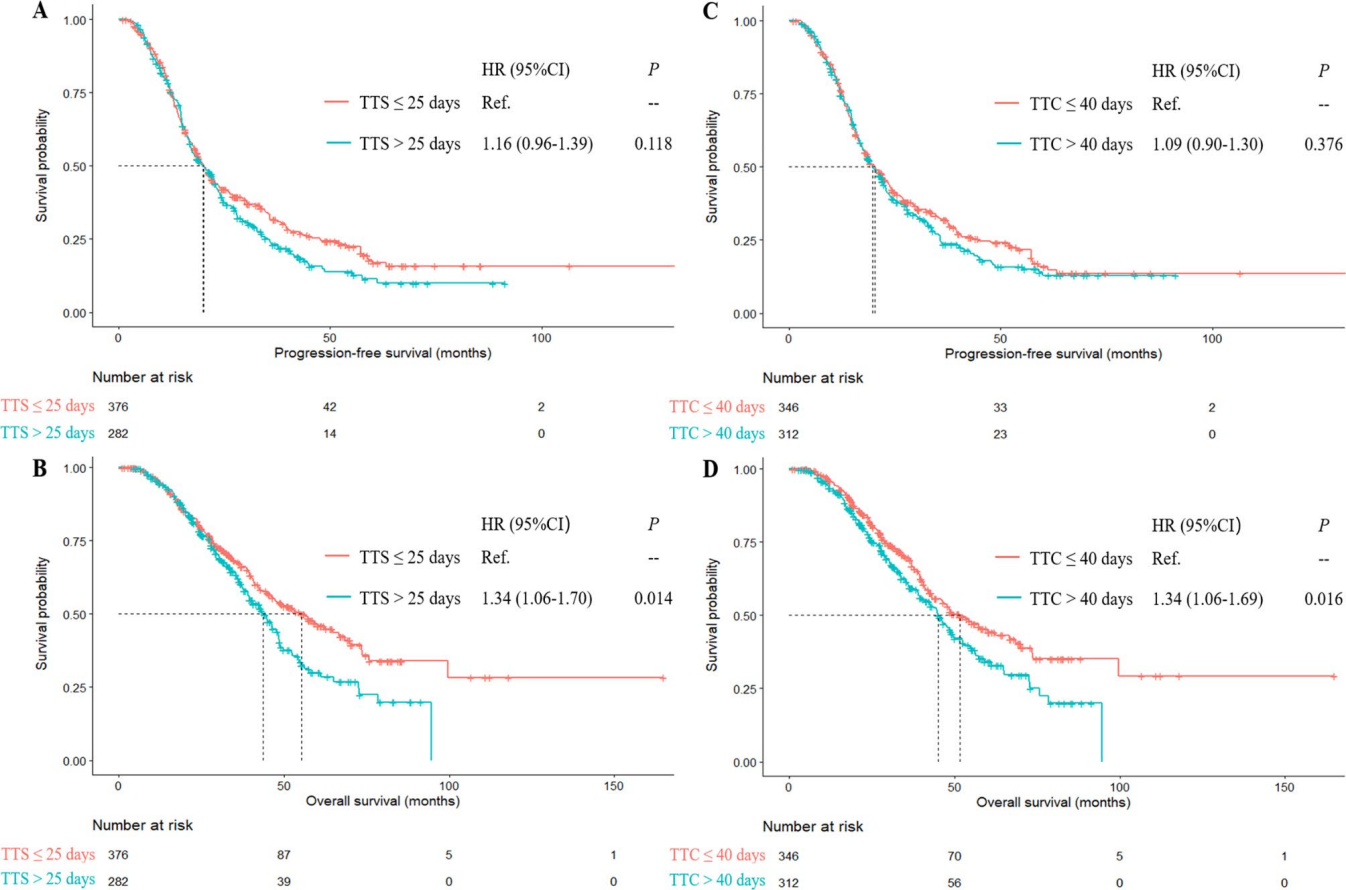
Survival analyses according to TTS and TTC. Figure **A** Kaplan–Meier curves of progression-free survival (**A**) of TTS. Figure **B**, Kaplan–Meier curves of overall survival (**B**) of TTS. Figure  **C**, Kaplan–Meier curves of progression-free survival (**C**) of TTC. Figure **D**, Kaplan–Meier curves of overall survival (**D**) of TTC. TTS, Time to interval debulking surgery after the completion of neoadjuvant chemotherapy; TTC, Time to postoperative adjuvant chemotherapy after the completion of neoadjuvant chemotherapy; HR, Hazard ratio; CI, Confidence interval

The median follow-up time for the 658 patients was 29.9 months (IQR, 19.7–45.1). The median follow-up time was 28.9 months (IQR, 19.1–48.0) for patients with TTS ≤ 25 days and 30.6 months (IQR, 21.4–43.0) for patients with TTS > 25 days (*P* = 0.962). The median PFS and OS of the overall patients were 20.1 months (95%CI, 18.3–21.9) and 47.8 months (95%CI, 43.2–52.4), respectively. The PFS was of no significant difference in patients with TTS ≤ 25 days and patients with TTS > 25 days (20.1 months vs. 19.9 months, *P* = 0.118, Fig. 1A). Patients with TTS > 25 days had a worse OS compared to the patients with TTS ≤ 25 days (55.2 months vs. 43.8 months, *P* = 0.014, Fig. 1B).

The results of univariate and multivariate Cox regression analyses of PFS and OS are presented in Table 2. In the multivariate analyses evaluating the impacts of several clinicopathological factors on PFS and OS, TTS > 25 days was not an independent risk factor for PFS (HR = 1.15, 95%CI: 0.96–1.39, *P* = 0.141) but was an independent risk factor for OS (HR = 1.34, 95%CI: 1.05–1.70, *P* = 0.018). Afterwards, the patients were divided into 4 groups according to the interquartile range of the TTS interval (≤ 20, 21–25, 26–29, > 29) (Table 3). After adjustment for other confounding factors, multivariate Cox regression revealed that TTS categorized by quartile values had a strong association with OS (*P* = 0.169, 0.014, 0.027, respectively) and patients with a longer TTS had a significantly higher risk of death (*P*_*trend*_ = 0.016). However, TTS had a weak association with PFS (*P* = 0.283, 0.115, 0.125, respectively) and the recurrence risk trend was not significantly different in TTS (*P*_*trend*_ = 0.103). Finally, TTS was an independent risk factor for OS but not associated with PFS when integrated as a continuous variable in the multivariate analyses (*P* = 0.018 and 0.228, respectively). Older age was associated with the delays of TTS (OR = 1.52, *P* = 0.019, Supplementary Table S4).

**Table 2:** Univariate and multivariate analyses for PFS and OS based on TTS

	PFS				OS			
	Univariate analysis		Multivariate analysis		Univariate analysis		Multivariate analysis	
	HR (95%CI)	*P* value	HR (95%CI)	*P* value	HR (95%CI)	*P* value	HR (95%CI)	*P* value
**Age, years**								
< 54	1 (Reference)		1 (Reference)		1 (Reference)		1 (Reference)	
≥ 54	1.02 (0.85–1.23)	0.827	0.98 (0.81–1.18)	0.804	1.18 (0.93–1.49)	0.175	1.10 (0.86–1.40)	0.440
**Type**								
Serous	1 (Reference)		1 (Reference)		1 (Reference)		1 (Reference)	
Other	1.24 (0.96–1.60)	0.098	0.89 (0.68–1.18)	0.421	1.15 (0.84–1.59)	0.385	0.93 (0.66–1.31)	0.679
**Grade**								
Other	1 (Reference)		1 (Reference)		1 (Reference)		1 (Reference)	
High	1.25 (0.98–1.60)	0.077	1.15 (0.88–1.51)	0.301	1.06 (0.78–1.44)	0.709	0.99 (0.71–1.37)	0.947
**FIGO Stage**								
IIIC	1 (Reference)		1 (Reference)		1 (Reference)		1 (Reference)	
IV	0.97 (0.80–1.17)	0.741	0.86 (0.70–1.05)	0.139	1.01 (0.79–1.3)	0.935	0.91 (0.71–1.18)	0.483
**Residual disease**								
R0	1 (Reference)		1 (Reference)		1 (Reference)		1 (Reference)	
R1	1.52 (1.24–1.87)	< 0.001	1.58 (1.28–1.95)	< 0.001	1.52 (1.16–1.99)	0.002	1.55 (1.17–2.04)	0.002
R2	1.61 (1.25–2.08)	< 0.001	1.69 (1.30–2.20)	< 0.001	1.89 (1.37–2.59)	< 0.001	1.99 (1.43–2.76)	< 0.001
**NACT cycle**								
≤ 3	1 (Reference)		1 (Reference)		1 (Reference)		1 (Reference)	
> 3	1.08 (0.86–1.36)	0.509	1.17 (0.93–1.49)	0.187	1.06 (0.79–1.42)	0.714	1.12 (0.83–1.52)	0.448
**TTS**								
≤ 25	1 (Reference)		1 (Reference)		1 (Reference)		1 (Reference)	
> 25	1.16 (0.96–1.39)	0.118	1.15 (0.96–1.39)	0.141	1.34 (1.06–1.70)	0.014	1.34 (1.05–1.70)	0.018

Abbreviation: PFS, Progression-free survival; OS, Overall survival; FIGO, International Federation of Gynecology and Obstetrics; NACT, Neoadjuvant chemotherapy; TTS, Time to interval debulking surgery after the completion of neoadjuvant chemotherapy; HR, Hazard ratio; CI, Confidence interval

**Table 3:** Multiple models for PFS and OS based on TTS

	PFS		OS	
	HR (95%CI)	*P* value	HR (95%CI)	*P* value
**TTS** ^**a**^				
≤ 20	1 (Reference)		1 (Reference)	
21–25	1.15 (0.89–1.48)	0.283	1.27 (0.91–1.77)	0.169
26–29	1.24 (0.95–1.62)	0.115	1.56 (1.09–2.21)	0.014
> 29	1.24 (0.94–1.62)	0.125	1.49 (1.05–2.12)	0.027
**TTS** ^**b**^	1.003 (0.997–1.010)	0.288	1.010 (1.002–1.018)	0.018
***P *** **for trend** ^**c**^		0.103*		0.016*

Abbreviation: PFS, Progression-free survival; OS, Overall survival; TTS, Time to interval debulking surgery after the completion of neoadjuvant chemotherapy; HR, Hazard ratio; CI, Confidence interval; *, *P* value for *P*_*trend*_

^a^ Adjusted for age, type, grade, stage, residual disease, cycle of neoadjuvant chemotherapy and TTS (included as a quartile categorical variable)

^b^ Adjusted for age, type, grade, stage, residual disease, cycle of neoadjuvant chemotherapy and TTS (included as a continuous variable)

^c^ Adjusted for age, type, grade, stage, residual disease and cycle of neoadjuvant chemotherapy

### Survival analysis based on TTC

In the 312 (47.4%) patients with a delayed time to PACT after the completion of NACT (TTC > 40 days), 192 (61.5%) patients had a delayed IDS (TTS > 25 days) and 246 (78.8%) patients had a delayed PACT after IDS (TI > 13 days). In 126 (40.4%) patients, both a delayed IDS after the completion of NACT and a delayed PACT after IDS occurred.

The median follow-up time was 30.6 months (IQR, 21.1–44.2) for patients with TTC ≤ 40 days and 29.1 months (IQR, 19.1–45.5) for patients with TTC > 40 days (*P* = 0.278). The PFS was of no significant difference in patients with TTC ≤ 40 days and patients with TTC > 40 days (20.2 months vs. 19.8 months, *P* = 0.376, Fig. 1C). Patients with TTC > 40 days had a worse OS compared to patients with TTC ≤ 40 days (51.7 months vs. 45.1 months, *P* = 0.016, Fig. 1D). As shown in Table 4, the association of PFS and OS with variables including age, histological type, grade, FIGO stage, upper abdominal surgery, bowel resection, lymphadenectomy, residual disease, postoperative complication, NACT cycle and TTC was analyzed. In the multivariate Cox regression analyses, TTC > 40 days had a strong association with worse OS (HR = 1.34, 95% CI: 1.05–1.71, *P* = 0.018) but a weak association with PFS (HR = 1.07, 95% CI: 0.89–1.30, *P* = 0.469). TTC categorized by quartile values had a strong association with OS (*P* = 0.012, 0.001, 0.033, respectively) and patients with a longer TTC had a significant higher risk of death (*P*_*trend*_ = 0.031). However, TTC was not associated with PFS (*P* = 0.221, 0.227, 0.338, respectively) and the recurrence risk trend was not significantly different in TTC (*P*_*trend*_ = 0.381). Finally, TTC was an independent risk factor for OS but not significant in PFS when integrated as a continuous variable in the multivariate analyses (*P* = 0.011 and 0.333, respectively) (Table 5).

**Table 4:** Univariate and multivariate analyses for PFS and OS based on TTC

	PFS				OS			
	Univariate analysis		Multivariate analysis		Univariate analysis		Multivariate analysis	
	HR (95%CI)	*P* value	HR (95%CI)	*P* value	HR (95%CI)	*P* value	HR (95%CI)	*P* value
**Age, years**								
< 54	1 (Reference)		1 (Reference)		1 (Reference)		1 (Reference)	
≥ 54	1.02 (0.85–1.23)	0.827	0.94 (0.78–1.13)	0.509	1.18 (0.93–1.49)	0.175	1.04 (0.82–1.32)	0.749
**Type**								
Serous	1 (Reference)		1 (Reference)		1 (Reference)		1 (Reference)	
Other	1.24 (0.96–1.60)	0.098	1.15 (0.87–1.52)	0.326	1.15 (0.84–1.59)	0.385	1.09 (0.77–1.55)	0.613
**Grade**								
Other	1 (Reference)		1 (Reference)		1 (Reference)		1 (Reference)	
High	1.25 (0.98–1.60)	0.077	1.12 (0.85–1.47)	0.418	1.06 (0.78–1.44)	0.709	1.00 (0.72–1.39)	0.985
**FIGO Stage**								
IIIC	1 (Reference)		1 (Reference)		1 (Reference)		1 (Reference)	
IV	0.97 (0.80–1.17)	0.741	0.88 (0.71–1.07)	0.200	1.01 (0.79–1.30)	0.935	0.91 (0.71–1.18)	0.484
**Upper abdominal surgery**								
No	1 (Reference)		1 (Reference)		1 (Reference)		1 (Reference)	
Yes	1.29 (0.97–1.72)	0.083	1.27 (0.94–1.71)	0.113	0.82 (0.54–1.26)	0.377	0.80 (0.52–1.25)	0.328
**Bowel resection**								
No	1 (Reference)		1 (Reference)		1 (Reference)		1 (Reference)	
Yes	1.18 (0.91–1.54)	0.209	1.09 (0.83–1.43)	0.556	1.46 (1.06-2.00)	0.02	1.36 (0.98–1.87)	0.064
**Lymphadenectomy**								
No	1 (Reference)		1 (Reference)		1 (Reference)		1 (Reference)	
Yes	0.73 (0.60–0.87)	0.001	0.73 (0.60–0.88)	0.001	0.72 (0.57–0.91)	0.006	0.76 (0.59–0.97)	0.030
**Residual disease**								
R0	1 (Reference)		1 (Reference)		1 (Reference)		1 (Reference)	
R1	1.52 (1.24–1.87)	< 0.001	1.56 (1.26–1.92)	< 0.001	1.52 (1.16–1.99)	0.002	1.51 (1.14–1.99)	0.004
R2	1.61 (1.25–2.08)	< 0.001	1.55 (1.19–2.02)	0.001	1.89 (1.37–2.59)	< 0.001	1.89 (1.35–2.64)	< 0.001
**Postoperative complication**								
No	1 (Reference)		1 (Reference)		1 (Reference)		1 (Reference)	
Yes	1.26 (1.05–1.51)	0.014	1.17 (0.97–1.42)	0.100	1.44 (1.14–1.82)	0.003	1.28 (1.00-1.63)	0.050
**NACT cycle**								
≤ 3	1 (Reference)		1 (Reference)		1 (Reference)		1 (Reference)	
> 3	1.08 (0.86–1.36)	0.509	1.21 (0.95–1.53)	0.119	1.06 (0.79–1.42)	0.714	1.15 (0.85–1.56)	0.372
**TTC**								
≤ 40	1 (Reference)		1 (Reference)		1 (Reference)		1 (Reference)	
> 40	1.09 (0.90–1.3)	0.376	1.07 (0.89–1.30)	0.469	1.34 (1.06–1.69)	0.016	1.34 (1.05–1.71)	0.018

Abbreviation: PFS, Progression-free survival; OS, Overall survival; FIGO, International Federation of Gynecology and Obstetrics; NACT, Neoadjuvant chemotherapy; TTC, Time to postoperative adjuvant chemotherapy after the completion of neoadjuvant chemotherapy; HR, Hazard ratio; CI, Confidence interval

**Table 5:** Multiple models for PFS and OS based on TTC

	PFS		OS	
	HR (95%CI)	*P* value	HR (95%CI)	*P* value
**TTC** ^**a**^				
≤ 33	1 (Reference)		1 (Reference)	
34–40	1.18 (0.91–1.52)	0.221	1.57 (1.10–2.23)	0.012
41–49	1.18 (0.90–1.53)	0.227	1.85 (1.31–2.62)	0.001
> 49	1.15 (0.87–1.51)	0.338	1.51 (1.03–2.19)	0.033
**TTC** ^**b**^	1.003 (0.997–1.009)	0.333	1.009 (1.002–1.017)	0.011
***P*** **for trend**^**c**^		0.381*		0.031*

Abbreviation: PFS, Progression-free survival; OS, Overall survival; TTC, Time to postoperative adjuvant chemotherapy after the completion of neoadjuvant chemotherapy; HR, Hazard ratio; CI, Confidence interval; *, *P* value for *P*_*trend*_

^a^ Adjusted for age, type, grade, stage, upper abdominal surgery, bowel resection, lymphadenectomy, residual disease, postoperative complication, cycle of neoadjuvant chemotherapy and TTC (included as a quartile categorical variable)

^b^ Adjusted for age, type, grade, stage, upper abdominal surgery, bowel resection, lymphadenectomy, residual disease, postoperative complication, cycle of neoadjuvant chemotherapy and TTC (included as a continuous variable)

^c^ Adjusted for age, type, grade, stage, upper abdominal surgery, bowel resection, lymphadenectomy, residual disease, postoperative complication and cycle of neoadjuvant chemotherapy

We further explored the impact of time interval (TI) from IDS to the initiation of PACT on prognosis. In the univariate analyses, TI was of no significant difference in PFS and OS (HR = 0.91, 95% CI: 0.76–1.09, *P* = 0.303, Supplementary Fig. S2A; HR = 1.14, 95% CI: 0.90–1.44, *P* = 0.287, Supplementary Fig. S2B, respectively). In multivariate cox analyses, TI was not significantly associated with PFS and OS categorized by median (*P* = 0.247 and 0.299, respectively), quartile values (*P* = 0.472, 0.139, 0.413 and 0.603, 0.853, 0.181, respectively) or integrated as a continuous variable (*P* = 0.910 and 0.150, respectively) (Supplementary Table S5). Besides, trends in recurrence and death did not alter with augmented TI (*P*_*trend*_ = 0.415 and 0.116, respectively). The associations of clinical characteristics and TI are shown in Supplementary Table S6. Bowel resection, lymphadenectomy and ≥ 54 years were correlated with the delay of chemotherapy (*P* = 0.003, *P* < 0.001 and *P* = 0.026, respectively).

## Discussion

In this multicenter real-world study, we explored the prognostic value of the timing of IDS and PACT after the completion of NACT in patients with advanced ovarian cancer. Our study elucidated that delays of IDS and PACT were significantly associated with impaired overall survival, while no significant associations were observed with PFS. Besides, in our study, patients with delays in IDS and PACT after the completion of NACT were older and tended to receive more NACT cycles.

NACT-IDS is the alternative for patients with advanced ovarian cancer with extensive metastases and unresectable lesions [18]. After the publication of the results of the European Organization for Research and Treatment of Cancer 55,971 trial in 2010, the use of NACT has elevated over the past decade [10]. Currently, the protocol of the ongoing SUNNY trial in China recommends IDS within 4–6 weeks after the completion of NACT and recommends the initiation of postoperative chemotherapy as soon as possible after surgery [19]. However, whether the delays of IDS and PACT after the completion of NACT have prognostic relevance remains undetermined.

Previous studies on the timing of IDS have conflicting results. In a retrospective study with 220 advanced epithelial ovarian cancer patients, Yong et al. found that patients with a TTS > 25 days had a significantly worse OS (*P* = 0.026) but a similar PFS (*P* = 0.552) compared to patients with a TTS ≤ 25 days [11]. On the contrary, another study which enrolled 152 advanced epithelial ovarian cancer patients revealed that TTS > 4 weeks was an independent risk factor for PFS (HR = 1.81, 95%CI: 1.35–2.52, *P* = 0.002) but not associated with OS (HR = 1.24, 95%CI: 0.79–1.89, *P* = 0.231) [12]. Ying et al. found that after adjusting for other clinical variables including age, stage and complete gross resection, TTS > 6 weeks was not significantly associated with PFS (HR = 1.66, 95%CI: 0.8–3.4, *P* = 0.17) and OS (HR = 1.55, 95%CI: 0.97–2.5, *P* = 0.062) [13]. The previous findings are also conflicting in other malignant tumors. In breast cancer, some studies showed that IDS should be performed within 3–6 weeks of completion of NACT [20–22], while another study revealed that the timing of IDS was not significantly associated with prognosis [23]. In pancreatic cancer, it was reported that the delay in IDS was even associated with a better prognosis (> 12 weeks vs. ≤ 12 weeks, HR = 0.80, 95%CI: 0.65–0.99; *P* = 0.042) [24]. In our study with 658 advanced epithelial ovarian cancer patients, TTS > 25 days was significantly associated with the worse OS but not with PFS. Moreover, longer TTS was an unfavorable factor for a higher risk of death.

As for the timing of PACT after the completion of NACT in ovarian cancer, Yong et al. reported that patients who started PACT after 42 days of the end of NACT were significantly associated with worse OS (HR = 2.03, 95%CI: 1.16–3.54, *P* = 0.013) but not with PFS (HR = 1.41, 95%CI: 0.98–2.03, *P* = 0.063) [11], which is consistent with our results. They also found that longer time intervals between the completion of NACT and the initiation of PACT were related to a higher risk of recurrence and death (*P*_*trend*_ = 0.006 and *P*_*trend*_ < 0.001, respectively) [11]. However, in our study, longer TTC was significantly associated with a higher risk of death but not with recurrence. Gemma et al. found that TTC > 10 weeks was associated with worse OS (*P* = 0.002) but not with PFS (*P* = 0.104) in a retrospective study of 205 patients with stage III and IV high-grade serous ovarian cancer [25]. These studies and our study demonstrated the significant association between TTC and OS. The delays of IDS and PACT showed a tendency of impaired PFS. We speculated that the results might be limited by the sample size.

In clinical settings, lots of factors could lead to delay of adjuvant chemotherapy, such as clinician preference, older age, postoperative complications, complete debulking surgery, and hospitalization days [26]. There might be some administrative reasons that delay the IDS or initiation date of PACT. The 7 hospitals included in our study are tertiary referral centers and the medical resources are relatively scarce, so the waiting time in line is relatively long. Given that our study period covered Jun 20, the regional quarantine and blockade policy and medical resources panic squeeze due to the COVID pandemic might lead to the delay. Some experimental studies demonstrated that tumor resection accelerated the growth of remaining lesions and perioperative chemotherapy had a significant advantage in hindering tumor recurrence [27–29]. However, whether TI could influence prognosis remains conflicting [17, 26]. In our study, TI was not significantly associated with survival in the multivariate analysis no matter how it entered the analysis. More real-world studies with a larger sample size were needed to explore the effects of TI on PFS and OS.

The rate of lymphadenectomy during IDS was relatively high in our study, which might due to the long-time span of the study and was similar to the results of other studies (46.7%~65.1%) [12, 30]. The median hospitalization of 16 days was relatively long in our study, compared with the median hospitalization of 7–10 days in other studies [26, 31]. One possible explanation of this was that a total of 348 (52.9%) patients completed the first cycle of PACT during their surgical hospitalization. Additionally, the time interval from admission to surgery (median, 5 days; IQR, 3–6) was relatively long in our study due to the various pre-operative examinations and evaluations. The median number of cycles of NACT was 2 in our study. Currently, 3–4 cycles of NACT followed by IDS was recommended by NCCN guidelines [4]. However, in our study and some previous studies of China, the median number of NACT cycles in advanced ovarian cancer was 2 [32–34]. The possible reasons for this might be the financial reasons and the recovery from inoperable medical conditions after 1–2 cycles of NACT. Recently, a prospective, multicentre, open-label, randomized phase III trial is being conducted to evaluate the efficacy and safety of patients receiving three (control group) or two cycles of NACT (experimental group) [35]. The number of NACT cycles has become a controversial hotspot [36, 37].

The strengths of this study include multicenter data, which allowed benchmarking among centers. Besides, the relatively large sample size significantly benefited the multivariate analysis of various clinical factors. In addition, we investigated TTS, TTC, and TI in the multivariate analyses by the median, quartile, and continuous variable and evaluated *P*_*trend*_, which guaranteed reliability of results. Finally, we have complete data with a relatively long follow-up period.

There are several limitations in this study. Due to its retrospective nature, there might have been some selection bias. Besides, since all data were derived from electronic medical records, this study is dependent on the accuracy and reliability of the electronic medical records. All the patients in our study are from tertiary hospitals, and the quality of medical records is relatively higher than in other centers of our country.

## Conclusions

The results of this study demonstrated that longer TTS and TTC are associated with worse OS. The delays of IDS and PACT after the completion of NACT may need to be avoided. Still, larger real-world studies are warranted to replicate our findings.

## Methods

### Study population

We performed the multicenter real-world retrospective analysis among patients with a histologically confirmed diagnosis of International Federation of Gynecology and Obstetrics (FIGO) stage IIIC-IV (2014 edition [38]) epithelial ovarian cancer who were admitted to the seven tertiary referral centers in China (detailed information showed in Supplementary Table S1) from June 2008 to June 2020. The study was approved by the Ethics Committee of Tongji Hospital of Huazhong University of Science and Technology and informed consent was waived (2020-S337).

All patients were clinically diagnosed with FIGO stage III or IV by computed tomography, magnetic resonance imaging and/or positron emission tomography/computed tomography. The diagnosis was histologically confirmed by image-biopsy or laparoscopic samples, or cytologically confirmed by ascites or pleural effusion. NACT was recommended by a multidisciplinary team consisted of two gynecological oncologists, one pathologist, one radiologist, and one medical oncologist if one of the following 2 criteria was met: (1) a medically inoperable condition and a high perioperative risk, and/or (2) optimal cytoreduction would not be achieved due to a high tumor burden. All patients were administered at least one cycle of NACT. The lymphadenectomy was performed if one of the following criteria was met: (1) Preoperative imaging evidence of enlarged lymph nodes or lymph node metastases, systemic lymphadenectomy or debulking of suspicious lymph node was performed depending on the discretion of surgeon or (2) enlarged lymph nodes was observed during IDS, systemic lymphadenectomy or debulking of suspicious lymph node was performed depending on the discretion of surgeon or (3) if optimal resection could be achieved, systemic lymphadenectomy was performed depending on the patient comorbidities and extent of surgery and the surgeon. A total of 92.7% of patients underwent systematic lymphadenectomy and 7.3% of patients underwent debulking of enlarged lymph node in our study.

Inclusion criteria were as follows: (1) histologically confirmed invasive epithelial ovarian, primary peritoneal, and fallopian tube cancers; (2) FIGO stage IIIC-IV; (3) treatment with NACT plus IDS and PACT; (4) known date of the completion of NACT, IDS and the initiation of PACT. Exclusion criteria were as follows: (1) borderline ovarian tumor; (2) peritoneal cancer that was not Müllerian origin, including mucinous histology; (3) no treatment with IDS or PACT; (4) unknown date of the completion of NACT, IDS or the initiation of PACT. In our study, 84 patients were excluded for the unknown date of the completion of NACT and 29 patients were excluded for the unknown date of initiation of PACT. In China, bevacizumab and poly (ADP-ribose) polymerase inhibitor (PARPI) are expensive and have been covered by the universal public social health insurance system only since 2020. In this study, 35 patients (5.3%) used bevacizumab and 17 patients (2.5%) used PARPI in the first-line treatment.

### Data collection

All the clinical data were extracted from the National Union of Real-world Gynecological Oncology Research and Patient Management platform, which was initiated by the National Clinical Research Center for Obstetrics and Gynecology in 2019, and integrated inpatient/outpatient clinical data, gene data, and follow-up information. Data were extracted from electronic medical records through the big data platform, then processed, standardized and structured according to pre-defined data sites. To guarantee the quality, data were cross-checked by two independent investigators.

Comorbidities included hypertension, cardiovascular and cerebrovascular diseases, diabetes, chronic liver disease, chronic lung disease, chronic kidney disease, and so on. Postoperative complication within 30 days after surgery was defined according to the Clavien-Dindo Classification [39]. As for residual disease, R0 was defined as no macroscopic residual disease; R1 was defined as the maximum diameter of postoperative residual disease ≤ 1 cm; R2 was defined as the maximum diameter of postoperative residual disease > 1 cm.

The time to interval debulking surgery (TTS) was defined as the time interval from the completion of NACT to the time of IDS. The time to postoperative adjuvant chemotherapy (TTC) was defined as the time interval from the completion of NACT to the initiation of PACT. The time interval (TI) was defined as the period from the time of IDS to the initiation of PACT (Supplementary Fig. S1).

Progression-free survival (PFS) was defined as the time interval between the time of diagnosis and the time of recurrence or progression or death or the last follow-up, whichever occurred first. Overall survival (OS) was defined as the time interval between the date of diagnosis and the date of death due to any course or the date of last follow-up.

### Statistical analysis

Continuous variables were described as median (interquartile ranges, IQR), and categorical variables were described as absolute values and percentages (%). The comparison of clinical characteristics was evaluated using the Chi-square test or Fisher’s exact test for categorical variables and the Student’s *t* test or an ANOVA test for continuous variables. Survival times and risk tables were analyzed with the Kaplan–Meier method and the log-rank test was performed to evaluate the statistical differences. Cox proportional hazard ratio (HR) and confidence interval (CI) was utilized to compare the survival data. The *P*_*trend*_ was evaluated by entering the median value of each group categorized by quartile of TTS, TTC and TI as continuous variables. All clinically significant factors were included in the multivariate analysis. All statistical tests were 2-tailed, and statistical significance was considered at *P* < 0.05. Kaplan–Meier survival curves were performed with the “survival” package in R software (version 4.05, R Foundation for Statistical Computing, Vienna, Austria). All other analyses were performed with SPSS statistical software (version 26.0, SPSS, Inc., Chicago, IL).

## Electronic supplementary material

Below is the link to the electronic supplementary material.

Additional file 1: **Supplementary Table S1.** List of Study Sites. **Supplementary table S2.** Clinical characteristics of patients according to TTS. Abbreviation: IQR, Interquartile range; BMI, Body mass index; FIGO, International Federation of Gynecology and Obstetrics; CA125, Cancer antigen 125; NACT, Neoadjuvant chemotherapy; Total cycles, the total number of cycles of both neoadjuvant chemotherapy and postoperative adjuvant chemotherapy; TTS, Time to interval debulking surgery after the completion of NACT. **Supplementary table S3.** Clinical characteristics of patients according to TTC. Abbreviation: IQR, Interquartile range; BMI, Body mass index; FIGO, International Federation of Gynecology and Obstetrics; CA125, Cancer antigen 125; NACT, Neoadjuvant chemotherapy; Total cycles, the total number of cycles of both neoadjuvant chemotherapy and postoperative adjuvant chemotherapy; TTC, Time to postoperative adjuvant chemotherapy after the completion of neoadjuvant chemotherapy. **Supplementary table S4.** Univariate and multivariate logistic regression analyses for delay of TTS. Abbreviation: TTS, Time to interval debulking surgery after the completion of neoadjuvant chemotherapy; OR, Odds ratio; CI, Confidence interval; BMI, Body mass index; FIGO, International Federation of Gynecology and Obstetrics. **Supplementary table S5.** Multiple models for PFS and OS based on TI Abbreviation: PFS, Progression-free survival; OS, Overall survival; TI, Time interval from interval debulking surgery to the initiation of postoperative adjuvant chemotherapy; HR, Hazard ratio; CI, Confidence interval; *, *P* value for *P*_*trend*_. ^a^ Adjusted for age, type, grade, stage, residual disease, cycle of neoadjuvant chemotherapy and TI (included as a binary variable). ^b^ Adjusted for age, type, grade, stage, residual disease, cycle of neoadjuvant chemotherapy and TI (included as a quartile categorical variable). ^c^ Adjusted for age, type, grade, stage, residual disease, cycle of neoadjuvant chemotherapy and TI (included as a continuous variable). ^d^ Adjusted for age, type, grade, stage, residual disease and cycle of neoadjuvant chemotherapy. **Supplementary table S6.** Univariate and multivariate logistic regression analyses for delay of PACT. Abbreviation: PACT, Postoperative adjuvant chemotherapy; OR, Odds ratio; CI, Confidence interval; FIGO, International Federation of Gynecology and Obstetrics. **Supplementary fig. S1.** Definitions of different time intervals. NACT, Neoadjuvant chemotherapy; IDS, Interval debulking surgery; PACT, Post-operative adjuvant chemotherapy; TTS, Time to interval debulking surgery after the completion of neoadjuvant chemotherapy; TTC, Time to postoperative adjuvant chemotherapy after the completion of neoadjuvant chemotherapy; TI, Time interval from interval debulking surgery to the initiation of postoperative adjuvant chemotherapy. **Supplementary fig. S2.** Survival analyses according to TI. Supplementary Fig. S2A, Kaplan-Meier curves of progression-free survival of TI. Supplementary Fig. S2B, Kaplan-Meier curves of overall survival of TI. TI, Time interval from interval debulking surgery to the initiation of postoperative adjuvant chemotherapy

## Data Availability

The datasets used and/or analysed during the current study are available from the corresponding author on reasonable request.

## Abbreviations

BMI: Body mass index
CA125: Cancer antigen 125
CI: Confidence interval
FIGO: International Federation of Gynecology and Obstetrics
HR: Hazard ratio
IDS: Interval debulking surgery
IQR: Interquartile range
NACT: Neoadjuvant chemotherapy
PACT: Postoperative adjuvant chemotherapy
PDS: Primary debulking surgery
RCT: Randomized controlled trial
TI: Time interval from surgery to chemotherapy
TTC: Time to postoperative adjuvant chemotherapy
TTS: Time to Interval debulking surgery
