# Supplementary material for: Timing of interval debulking surgery and postoperative chemotherapy after neoadjuvant chemotherapy in advanced epithelial ovarian cancer: a multicenter real-world study
Source: J Ovarian Res. 2023 Jun 27;16:121. doi: 10.1186/s13048-023-01164-8 (PMC10294495; doi:10.1186/s13048-023-01164-8)
Supplement: Supplementary file 1 — Additional file 1: Supplementary Table S1. List of Study Sites. Supplementary table S2. Clinical characteristics of patients according to TTS. Abbreviation: IQR, Interquartile range; BMI, Body mass index; FIGO, International Federation of Gynecology and Obstetrics; CA125, Cancer antigen 125; NACT, Neoadjuvant chemotherapy; Total cycles, the total number of cycles of both neoadjuvant chemotherapy and postoperative adjuvant chemotherapy; TTS, Time to interval debulking surgery after the completion of NACT. Supplementary table S3. Clinical characteristics of patients according to TTC. Abbreviation: IQR, Interquartile range; BMI, Body mass index; FIGO, International Federation of Gynecology and Obstetrics; CA125, Cancer antigen 125; NACT, Neoadjuvant chemotherapy; Total cycles, the total number of cycles of both neoadjuvant chemotherapy and postoperative adjuvant chemotherapy; TTC, Time to postoperative adjuvant chemotherapy after the completion of neoadjuvant chemotherapy. Supplementary table S4. Univariate and multivariate logistic regression analyses for delay of TTS. Abbreviation: TTS, Time to interval debulking surgery after the completion of neoadjuvant chemotherapy; OR, Odds ratio; CI, Confidence interval; BMI, Body mass index; FIGO, International Federation of Gynecology and Obstetrics. Supplementary table S5. Multiple models for PFS and OS based on TI Abbreviation: PFS, Progression-free survival; OS, Overall survival; TI, Time interval from interval debulking surgery to the initiation of postoperative adjuvant chemotherapy; HR, Hazard ratio; CI, Confidence interval; *, P value for Ptrend. a Adjusted for age, type, grade, stage, residual disease, cycle of neoadjuvant chemotherapy and TI (included as a binary variable). b Adjusted for age, type, grade, stage, residual disease, cycle of neoadjuvant chemotherapy and TI (included as a quartile categorical variable). c Adjusted for age, type, grade, stage, residual disease, cycle of neoadjuvant chemotherapy and TI (i [file 13048_2023_1164_MOESM1_ESM.docx]

**Supplementary Online Content**

**Supplementary Table S1. List of Study Sites.**

**Supplementary Table S2. Clinical characteristics of patients according to TTS.**

**Supplementary Table S3. Clinical characteristics of patients according to TTC.**

**Supplementary Table S4. Univariate and multivariate logistic regression analyses for delay of TTS.**

**Supplementary Table S5. Multiple models for PFS and OS based on TI.**

**Supplementary Table S6. Univariate and multivariate logistic regression analyses for delay of PACT.**

**Supplementary Fig. S1. Definitions of different time intervals.**

**Supplementary Fig. S2. Survival analyses according to TI.**

**Supplementary Table S1. List of Study Sites.**

| **No.** | **Study Sites** |
| --- | --- |
| 1 | Tongji Hospital affiliated to Tongji Medical College of Huazhong University of Science and Technology |
| 2 | The First Affiliated Hospital of Sun Yat-sen University |
| 3 | The First Affiliated Hospital of Soochow University |
| 4 | Henan Cancer Hospital |
| 5 | Shandong Cancer Hospital |
| 6 | Hunan Cancer Hospital |
| 7 | Chongqing Cancer Hospital |

**Supplementary Table S2.** **Clinical characteristics of patients according to TTS.**

|  | **TTS ≤ 25 days (N=376)** | **TTS > 25 days (N=282)** | ***P* value** |
| --- | --- | --- | --- |
| **Age (years, median (IQR))** | 53.00 (47.00, 61.00) | 55.00 (49.00, 62.00) | 0.012 |
| **Menopause** | | | 0.113 |
| Yes | 223 (59.3) | 183 (64.9) |  |
| No | 137 (36.4) | 94 (33.3) |  |
| Unknown | 16 (4.3) | 5 (1.8) |  |
| **BMI (kg/m^2^, median (IQR))** | 22.62 (20.45, 24.65) | 22.43 (20.73, 24.46) | 0.639 |
| **Comorbidity** | | | 0.897 |
| Yes | 129 (34.3) | 99 (35.1) |  |
| No | 247 (65.7) | 183 (64.9) |  |
| **Type** | | | 0.567 |
| Serous | 314 (83.5) | 241 (85.5) |  |
| Other | 62 (16.5) | 41 (14.5) |  |
| **Grade** | | | 0.747 |
| High | 318 (84.6) | 235 (83.3) |  |
| Other | 58 (15.4) | 47 (16.7) |  |
| **FIGO stage** | | | 0.647 |
| IIIC | 245 (65.2) | 178 (63.1) |  |
| IV | 131 (34.8) | 104 (36.9) |  |
| **CA125 (U/ml, median (IQR))** | 1302.70 (600.00, 3039.30) | 1402.35 (712.42, 3349.02) | 0.354 |
| **Ascites** | | |  |
| Yes | 306 (81.4) | 231 (81.9) |  |
| No | 68 (18.1) | 46 (16.3) | 0.268 |
| Unknown | 2 (0.5) | 5 (1.8) |  |
| **Residual disease** | | | 0.792 |
| R0 | 173 (46.0) | 124 (44.0) |  |
| R1 | 133 (35.4) | 107 (37.9) |  |
| R2 | 70 (18.6) | 51 (18.1) |  |
| **NACT cycles (median (IQR))** | 2 (2, 3) | 3 (2, 3) | 0.002 |
| **Chemotherapy regimen** | | | 0.833 |
| Platinum + Paclitaxel | 370 (98.4) | 276 (97.9) |  |
| Other | 6 (1.6) | 6 (2.1) |  |
| **Total cycles (median (IQR))** | 7 (6, 9) | 7 (6, 9) | 0.507 |

Abbreviation: IQR, Interquartile range; BMI, Body mass index; FIGO, International Federation of Gynecology and Obstetrics; CA125, Cancer antigen 125; NACT, Neoadjuvant chemotherapy; Total cycles, the total number of cycles of both neoadjuvant chemotherapy and postoperative adjuvant chemotherapy; TTS, Time to interval debulking surgery after the completion of NACT.

**Supplementary Table S3. Clinical characteristics of patients according to TTC.**

|  | **TTC ≤ 40 days (N=346)** | **TTC > 40 days (N=312)** | ***P* value** |
| --- | --- | --- | --- |
| **Age (years, median (IQR))** | 53.00 (47.00, 60.00) | 55.00 (48.00, 62.00) | 0.022 |
| **Menopause** | | | 0.043 |
| Yes | 198 (57.2) | 208 (66.7) |  |
| No | 135 (39.0) | 96 (30.8) |  |
| Unknown | 13 (3.8) | 8 (2.6) |  |
| **BMI (kg/m^2^, median (IQR))** | 22.66 (20.78, 25.20) | 22.35 (20.40, 24.18) | 0.054 |
| **Comorbidity** | | | 0.669 |
| Yes | 123 (35.5) | 105 (33.7) |  |
| No | 223 (64.5) | 207 (66.3) |  |
| **Type** | | | 0.431 |
| Serous | 296 (85.5) | 259 (83.0) |  |
| Other | 50 (14.5) | 53 (17.0) |  |
| **Grade** | | | 0.563 |
| High | 294 (85.0) | 259 (83.0) |  |
| Other | 52 (15.0) | 53 (17.0) |  |
| **FIGO Stage** | | | 0.861 |
| IIIC | 224 (64.7) | 199 (63.8) |  |
| IV | 122 (35.3) | 113 (36.2) |  |
| **CA125 (U/ml, median (IQR))** | 1585.90 (679.90, 3580.50) | 1213.85 (627.38, 2899.00) | 0.234 |
| **Ascites** | | | 0.900 |
| Yes | 284 (82.1) | 253 (81.1) |  |
| No | 58 (16.8) | 56 (17.9) |  |
| Unknown | 4 (1.2) | 3 (1.0) |  |
| **Operation time (mins, median (IQR))** | 188.50 (145.00, 249.75) | 230.00 (175.00, 310.00) | <0.001 |
| **Blood loss (ml, median (IQR))** | 300.00 (200.00, 600.00) | 300.00 (200.00, 600.00) | 0.448 |
| **Surgical procedure** | | | |
| Upper abdominal surgery | 30 (8.7) | 45 (14.4) | 0.028 |
| Bowel resection | 39 (11.3) | 51 (16.3) | 0.075 |
| Lymphadenectomy | 162 (46.8) | 181 (58.0) | 0.005 |
| **Postoperative complication** | | | 0.010 |
| Yes | 158 (45.7) | 175 (56.1) |  |
| No | 188 (54.3) | 137 (43.9) |  |
| **Residual disease** | | | 0.403 |
| R0 | 161 (46.5) | 136 (43.6) |  |
| R1 | 118 (34.1) | 122 (39.1) |  |
| R2 | 67 (19.4) | 54 (17.3) |  |
| **Hospitalization (days, median (IQR))** | 16 (14, 19) | 17 (13, 22) | 0.195 |
| **NACT cycles (median (IQR))** | 2.00 (2.00, 3.00) | 3.00 (2.00, 3.00) | 0.154 |
| **Chemotherapy regimen** | | | 1.000 |
| Platinum + Paclitaxel | 340 (98.3) | 306 (98.1) |  |
| Other | 6 (1.7) | 6 (1.9) |  |
| **Total cycles (median (IQR))** | 7.00 (6.00, 8.00) | 7.00 (6.00, 9.00) | 0.740 |

Abbreviation: IQR, Interquartile range; BMI, Body mass index; FIGO, International Federation of Gynecology and Obstetrics; CA125, Cancer antigen 125; NACT, Neoadjuvant chemotherapy; Total cycles, the total number of cycles of both neoadjuvant chemotherapy and postoperative adjuvant chemotherapy; TTC, Time to postoperative adjuvant chemotherapy after the completion of neoadjuvant chemotherapy.

**Supplementary Table S4. Univariate and multivariate logistic regression analyses for delay of TTS.**

|  | **Univariate analysis** | | **Multivariate analysis** | |
| --- | --- | --- | --- | --- |
|  | OR (95%CI) | *P* value | OR (95%CI) | *P* value |
| **Age, years** |  |  |  |  |
| < 54 | 1 (Reference) |  | 1 (Reference) |  |
| ≥ 54 | 1.53 (1.08-2.15) | 0.016 | 1.52 (1.07-2.15) | 0.019 |
| **Comorbidity** |  |  |  |  |
| No | 1 (Reference) |  | 1 (Reference) |  |
| Yes | 1.08 (0.76-1.55) | 0.656 | 1.04 (0.72-1.50) | 0.836 |
| **BMI (kg/m^2^)** | 0.98 (0.93-1.04) | 0.548 | 0.98 (0.93-1.03) | 0.433 |
| **Ascites** |  |  |  |  |
| No | 1 (Reference) |  | 1 (Reference) |  |
| Yes | 1.35 (0.84-2.18) | 0.215 | 1.39 (0.86-2.26) | 0.180 |
| **FIGO stage** |  |  |  |  |
| IIIC | 1 (Reference) |  | 1 (Reference) |  |
| IV | 1.09 (0.77-1.56) | 0.622 | 1.13 (0.78-1.62) | 0.520 |

Abbreviation: TTS, Time to interval debulking surgery after the completion of neoadjuvant chemotherapy; OR, Odds ratio; CI, Confidence interval; BMI, Body mass index; FIGO, International Federation of Gynecology and Obstetrics.

**Supplementary Table S5. Multiple models for PFS and OS based on TI.**

|  | **PFS** | | **OS** | |
| --- | --- | --- | --- | --- |
|  | HR (95%CI) | *P* value | HR (95%CI) | *P* value |
| **TI^a^** |  |  |  |  |
| ≤ 13 | 1 (Reference) |  | 1 (Reference) |  |
| > 13 | 0.90 (0.75-1.08) | 0.247 | 1.14 (0.89-1.44) | 0.299 |
| **TI^b^** |  |  |  |  |
| ≤ 9 | 1 (Reference) |  | 1 (Reference) |  |
| 10-13 | 0.91 (0.70-1.18) | 0.472 | 0.91 (0.65-1.29) | 0.603 |
| 14-22 | 0.83 (0.64-1.06) | 0.139 | 0.97 (0.70-1.34) | 0.853 |
| > 22 | 0.90 (0.70-1.16) | 0.413 | 1.25 (0.90-1.73) | 0.181 |
| **TI^c^** | 0.999 (0.990-1.009) | 0.910 | 1.009 (0.997-1.020) | 0.150 |
| ***P* for trend^d^** |  | 0.415* |  | 0.116* |

Abbreviation: PFS, Progression-free survival; OS, Overall survival; TI, Time interval from interval debulking surgery to the initiation of postoperative adjuvant chemotherapy; HR, Hazard ratio; CI, Confidence interval; *, *P* value for *P_trend_*.

^a^ Adjusted for age, type, grade, stage, residual disease, cycle of neoadjuvant chemotherapy and TI (included as a binary variable).

^b^ Adjusted for age, type, grade, stage, residual disease, cycle of neoadjuvant chemotherapy and TI (included as a quartile categorical variable).

^c^ Adjusted for age, type, grade, stage, residual disease, cycle of neoadjuvant chemotherapy and TI (included as a continuous variable).

^d^ Adjusted for age, type, grade, stage, residual disease and cycle of neoadjuvant chemotherapy.

**Supplementary Table S6. Univariate and multivariate logistic regression analyses for delay of PACT.**

|  | **Univariate analysis** | | **Multivariate analysis** | |
| --- | --- | --- | --- | --- |
|  | OR (95%CI) | *P* value | OR (95%CI) | *P* value |
| **Age, years** |  |  |  |  |
| < 54 | 1 (Reference) |  | 1 (Reference) |  |
| ≥ 54 | 1.28 (0.94-1.74） | 0.117 | 1.44 (1.05-1.99) | 0.026 |
| **FIGO Stage** |  |  |  |  |
| IIIC | 1 (Reference) |  | 1 (Reference) |  |
| IV | 0.97 (0.71-1.34) | 0.871 | 1.00 (0.71-1.40) | 0.980 |
| **Residual disease** |  |  |  |  |
| R0 | 1 (Reference) |  | 1 (Reference) |  |
| R1 | 1.02 (0.73-1.43) | 0.907 | 1.04 (0.72-1.49) | 0.838 |
| R2 | 0.85 (0.56-1.30) | 0.454 | 0.96 (0.61-1.52) | 0.869 |
| **Upper abdominal surgery** |  |  |  |  |
| No | 1 (Reference) |  | 1 (Reference) |  |
| Yes | 1.22 (0.75-1.98) | 0.419 | 1.10 (0.66-1.83) | 0.709 |
| **Bowel resection** |  |  |  |  |
| No | 1 (Reference) |  | 1 (Reference) |  |
| Yes | 2.09 (1.31-3.31) | 0.002 | 2.08 (1.28-3.36) | 0.003 |
| **Lymphadenectomy** |  |  |  |  |
| No | 1 (Reference) |  | 1 (Reference) |  |
| Yes | 2.45 (1.79-3.35 | <0.001 | 2.58 (1.86-3.58) | <0.001 |
| **Postoperative complication** |  |  |  |  |
| No | 1 (Reference) |  | 1 (Reference) |  |
| Yes | 1.00 (0.74-1.36） | 0.995 | 1.02 (0.73-1.41) | 0.918 |

Abbreviation: PACT, Postoperative adjuvant chemotherapy; OR, Odds ratio; CI, Confidence interval; FIGO, International Federation of Gynecology and Obstetrics.


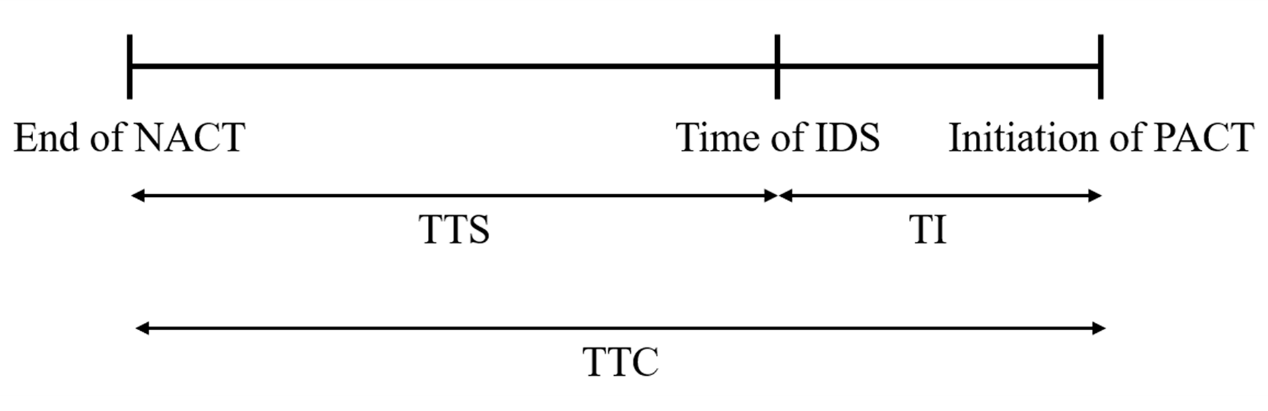


**Supplementary Fig. S1. Definitions of different time intervals.**

NACT, Neoadjuvant chemotherapy; IDS, Interval debulking surgery; PACT, Post-operative adjuvant chemotherapy; TTS, Time to interval debulking surgery after the completion of neoadjuvant chemotherapy; TTC, Time to postoperative adjuvant chemotherapy after the completion of neoadjuvant chemotherapy; TI, Time interval from interval debulking surgery to the initiation of postoperative adjuvant chemotherapy.


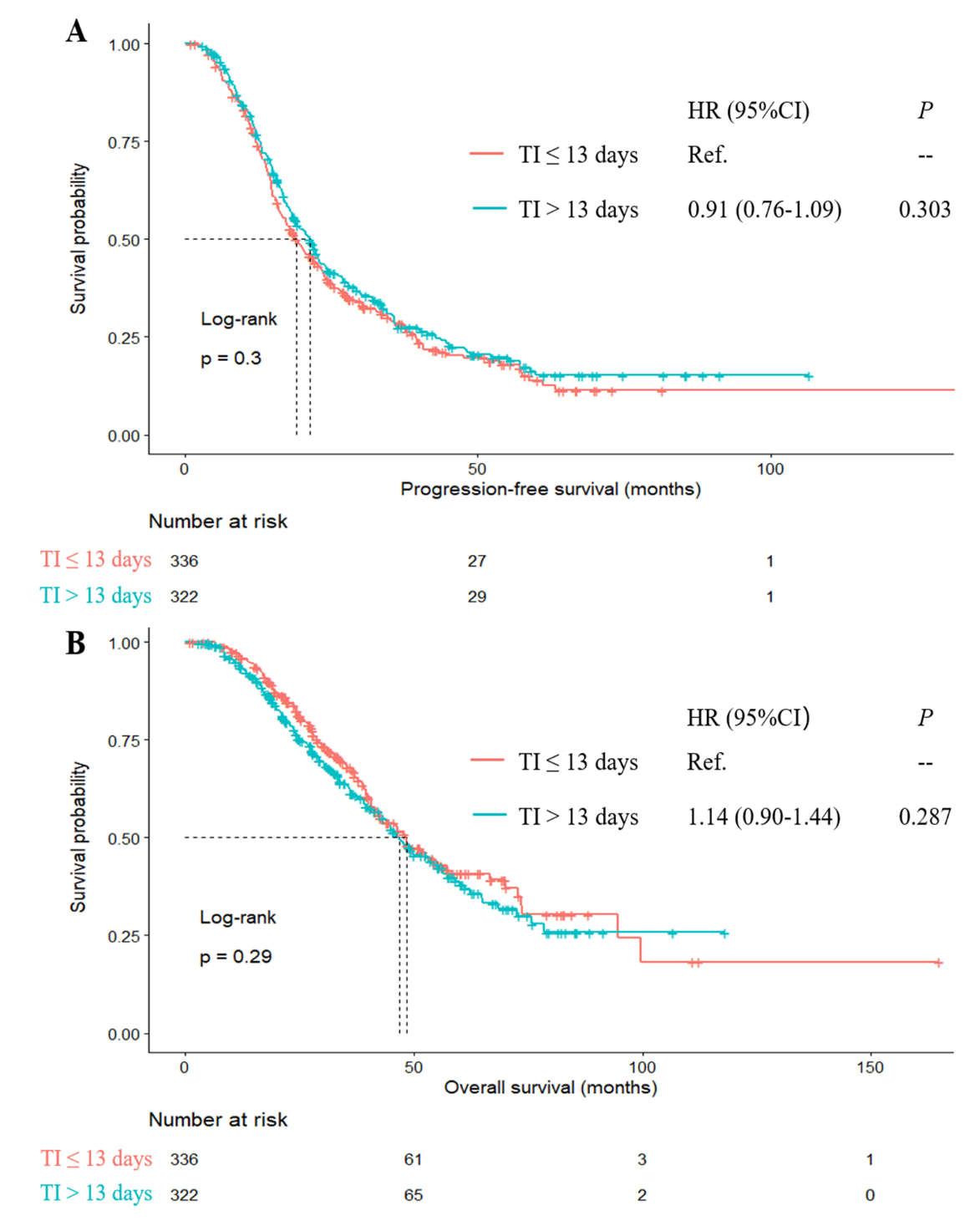


**Supplementary Fig. S2** **Survival analyses according to TI.**

Supplementary Fig. S2A, Kaplan-Meier curves of progression-free survival of TI. Supplementary Fig. S2B, Kaplan-Meier curves of overall survival of TI. TI, Time interval from interval debulking surgery to the initiation of postoperative adjuvant chemotherapy.
